# Updates in Assisted Reproduction

**DOI:** 10.3390/jcm11113129

**Published:** 2022-05-31

**Authors:** Charalampos Siristatidis, Kalliopi Syristatidi, Michail Papapanou

**Affiliations:** 1 Assisted Reproduction Unit, Second Department of Obstetrics and Gynecology, “Aretaieion” University Hospital, Medical School, National and Kapodistrian University of Athens, 76 Vas. Sofias Av., 11528 Athens, Greece; mixalhspap13@gmail.com; 2 School of Medicine, University of St. Andrews, North Haugh, St. Andrews KY16 9TF, UK; ksyristatidi@gmail.com; 3 Obstetrics, Gynecology and Reproductive Medicine Working Group, Society of Junior Doctors, 15123 Athens, Greece

**Keywords:** assisted reproductive techniques, in vitro fertilization

## Abstract

There are multiple reasons for which the “updates in assisted reproduction” topic is and will be in the center of scientific attention—both clinical and laboratory—during the next decades. In this editorial, we present and discuss some of them.

In the current Special Issue “Updates in assisted reproduction treatment”, three important papers have been published so far, addressing the following topics: the role of hysteroscopy prior to an in vitro fertilization (IVF) cycle [1], the type of anesthesia applied during oocyte retrieval [2], and a pre-embryo transfer endometrial procedure in frozen cycles [3]. The results of these papers, though somewhat different from existing notions, provide an additive character to the past literature that is of great interest.

There are various reasons for which the advances in assisted reproduction will remain both a compelling question and likely an unachievable target during the next—at least—three decades, with the first, and most important, being that assisted reproduction constitutes the only field in medicine involved in creating human life.

Secondly, assisted reproduction treatment remains inefficient. Quoting the introductory part of this Special Issue: “An estimated 8% to 12% of couples of reproductive age face challenges in achieving pregnancy within a year of regular, timed, and unprotected intercourse, with global surveys reporting as many as 186 million individuals suffering from infertility. For the past 40 years, assisted reproduction technology (ART) utilizes scientific knowledge and sophisticated technology for infertility management, even though compared with the degree of intervention, the success rates remain low, with only 30% of the embryos produced in vitro being ultimately transferred to the uterus, and only 10–30% of transferred embryos progressing to live birth. This realization calls for a fresh view on infertility management, along with the new perspectives on modern lifestyle and social structure that delays family planning. Practitioners are constantly seeking adjunct treatments to improve the outcomes, in the form of medical or non-medical co-therapies” [4,5]. Of note, the causes of low success rates in reproductive medicine still seem to be evident; the advanced age of women seeking IVF, the lack of effectiveness of “adds-on” to IVF in conjunction with the consolidation, industrialization, and commoditization of ART, consist of merely a part of these causes [6,7].

Thirdly, the quality of the studies published in the international literature are far from ideally providing robust conclusions and meaningfully guiding future research. In a recent paper [8], we addressed the potential flaws and pitfalls in research conduct, along with recommendations for the improvement of study designs/methods and scientific reporting. This aimed at promoting publication quality and stricter criteria for its release with support from the appropriate structures. Additionally, we emphasized the ethical need to “put patient-first”, as well as the need for facilitating a set goal via healthy network collaborations [8]. In another paper [9], the authors came to the same conclusion, reporting flaws in the ethics of data reporting, dysfunctional peer review, conflicts of interest in editorial offices, patient selection biases, and the utilization of incorrect statistical methodologies. In the same context, a constant and—difficult to resolve—issue of the relevant literature is the inconsistency between targeted populations and the employed methods [10]. Differences in patient backgrounds (i.e., age groups, patients’ underlying conditions and/or couples’ infertility factors and special characteristics) and IVF clinics’ equipment and implemented protocols (e.g., ovarian stimulation protocols, the conditions during embryo culture, the developmental stage of the embryo at time of transfer, the day of transfer within the same developmental stage, the number of embryo transfers, cryopreservation methods in case of frozen cycles) constitute only some of the illustrative examples of this clinical inconsistency [10,11,12,13,14,15,16]. Consequently, there exists a large variation between IVF studies, which often leads to the downgrading of the synthesized evidence due to inconsistency-related and confounding factors. As this variation remains and will continue to remain prevalent, unified international databases covering a broad spectrum of settings are required. The recording of predefined parameters could result in the adjustment for/identification of at least some of the potential confounding factors/effect modifiers. Considering that such a concept may, at this point, lack in feasibility and would therefore require the composition of an international collaborative and significant effort for design and materialization, original study investigators (especially trialists) are strongly encouraged to provide their individual participant data (IPD) to be used for IPD meta-analyses [17]. The latter may be achieved by either directly sharing the raw patient data with the meta-analysis study group or by making the data available via data-sharing repositories/platforms (and after acquiring the necessary permissions in compliance with General Data Protection Regulation) [17]. Although the IPD-meta-analysis design requires more dedicated time and resources than a conventional systematic review, it may partially, but more effectively than current aggregate-data approaches, address some of the abovementioned pitfalls of the ART literature [17,18].

Fourthly, there is an inexplicably high number of clinicians who perform ART having received insufficient training and possessing no appropriate certification. Although interventions concerning an IVF cycle are relatively easy to perform and demonstrate low complication rates [19], there exists a wider range of knowledge that a clinician must possess, such as, but most definitely not limited to, knowledge of continuous learning/research, ethics, and regulatory procedures/practices [20].

Promisingly, advances in assisted reproduction constantly emerge, reminding humankind that the scientific field involved in the creation of life is a rapidly evolving one. In a newly published paper [21], authors suggest such potential future steps. The study includes and utilizes new information on the origin of humans and human-specific functions (with a focus on (epi)genetics in development), and on the evolution of new tools and genome editing technologies, such as CRISPR/Cas9, and always within the appropriate ethical framework [21]. It also discusses the discovery of additional human-specific genetic features, molecular networks, cells, tissue types, an assessment of the safety and efficacy of the numerous applied biomedical approaches, and the extended culture of human embryos [21]. Finally, the authors highlight the improvement of in vitro primordial follicle culture and the cryopreservation methods of ovarian tissue, embryos, and oocytes, as well as new advances in the emerging field of embryo modeling, and in the ability to produce gametes in vitro [21].

In conclusion, ART, being a rapidly evolving combination of science and technology that is dedicated to the creation of human life, will probably remain in the scientific spotlight for decades to come. Establishing a fresh and couple-centered view on infertility management that accords with modern lifestyle-related factors, improving the flaws of current practices and the relevant literature, and addressing the apparent inconsistency between studied populations and settings are all necessary components of the continuous effort towards the improvement of the employed methods’ effectiveness, and thus the achievement of better outcomes.

## Data Availability

Not applicable.
